# Ramucirumab plus FOLFIRI or irinotecan as second-line treatment for patients with gastroesophageal adenocarcinoma: a review and meta-analysis of an emerging option

**DOI:** 10.3389/fonc.2024.1419338

**Published:** 2024-08-13

**Authors:** Haeseong Park, Samuel J. Klempner, Joseph Chao, Zev A. Wainberg, Mariusz Lukanowski, Suresh Chenji, Shannon Bourke, Anindya Chatterjee, Sylvie Lorenzen

**Affiliations:** ^1^ Department of Medicine, Gastrointestinal Cancer Center, Dana Farber Cancer Institute, Harvard Medical School, Boston, MA, United States; ^2^ Department of Medicine, Massachusetts General Hospital, Boston, MA, United States; ^3^ Department of Medical Oncology & Therapeutics Research, City of Hope, Duarte, CA, United States; ^4^ Gastrointestinal Oncology Program, Department of Medicine, Division of Hematology/Oncology, University of California, Los Angeles (UCLA), Los Angeles, CA, United States; ^5^ Oncology Medical Affairs, Eli Lilly and Company, Indianapolis, IN, United States; ^6^ Global Statistical Sciences, Eli Lilly and Company, Indianapolis, IN, United States; ^7^ Department of Oncology Eli Lilly and Company, Indianapolis, IN, United States; ^8^ Global Medical Affairs, Oncology, Eli Lilly and Company, Indianapolis, IN, United States; ^9^ Third Department of Internal Medicine (Hematology/Medical Oncology), Technical University of Munich, Munich, Germany

**Keywords:** ramucirumab, gastroesophageal adenocarcinoma, second-line, irinotecan, FOLFIRI

## Abstract

**Introduction:**

The aim of this study was to provide a review of the clinical evidence for use of ramucirumab (RAM) plus folinic acid (leucovorin), fluorouracil (5-FU), and irinotecan (FOLFIRI) or irinotecan as second-line treatment in gastroesophageal adenocarcinoma (GEA).

**Methods:**

A systematic and comprehensive search of PubMed was performed to identify phase 2 clinical trials or retrospective studies using RAM plus FOLFIRI or irinotecan in GEA, including abstracts from major congresses, in addition to published manuscripts. An aggregated review and meta-analysis was performed to assess the effectiveness (overall response rate [ORR] as primary outcome) and safety data of RAM plus FOLFIRI or irinotecan. ORR for each study was calculated with 95% confidence interval estimated from normal approximation. To generate the combined ORR with 95% confidence interval, random-effects meta-analysis was conducted to synthesize response data from available studies.

**Results:**

Six studies were identified with non-overlapping populations, 3 phase 2 clinical trials and 3 retrospective studies. Across these studies the ORR ranged from 22% to 38%, and pooled ORR was 25.4%. Two of the 3 studies reported better ORR in patients pretreated with taxanes followed by RAM plus FOLFIRI. Treatment with RAM plus FOLFIRI or irinotecan was well tolerated. Neutropenia and diarrhea were the most common adverse events reported across studies.

**Conclusion:**

The studies examined in this review suggest that RAM plus FOLFIRI or irinotecan have activity in previously treated GEA irrespective of prior-taxane use. Overall, RAM plus FOLFIRI or irinotecan was well tolerated with no new safety concerns identified beyond established profiles for these regimens.

## 1 Introduction

Gastroesophageal adenocarcinomas (GEA) account for over 1.3 million annual deaths, representing nearly 13.2% of global cancer deaths (1). In the past few years, clinical advances including upfront use of programmed cell death protein 1 (PD-1) inhibitors and the addition of docetaxel in the perioperative treatment may impact survival benefits across lines of therapy in advanced GEA (2). The current standard first-line treatment for advanced GEA is platinum and fluoropyrimidine-based doublet with or without the addition of a PD-1 inhibitor and with trastuzumab in human epidermal growth factor receptor 2-positive disease (3, 4). Taxane-containing regimens used in localized and advanced disease is increasing, and perioperative chemotherapy with fluorouracil, leucovorin, oxaliplatin, and docetaxel became standard in many regions (2). In Japan, S1 (a novel oral fluoropyrimidine derivative) plus docetaxel is the new standard of care for the adjuvant therapy of stage III gastric cancer (5).

Despite such advances in first-line and perioperative treatments, there is no randomized phase 3 trial that improves upon ramucirumab (RAM) plus paclitaxel in the later lines of treatment. RAM with paclitaxel has been established as second-line standard after platinum and fluoropyrimidine-containing treatment on the basis of positive results of the phase 3 RAINBOW trial (6). RAM is a recombinant human immunoglobulin G1 monoclonal antibody receptor antagonist designed to bind to the extracellular domain of vascular endothelial growth factor receptor 2, thereby blocking the binding of multiple vascular endothelial growth factor (VEGF) ligands and inhibiting receptor activation (7). Also, ramucirumab inhibits all VEGFs thus enabling inhibition of downstream receptor activation of VEGF signaling pathways resulting in reduced tumor neovascularization and growth (8). Chemotherapy in combination with anti-vascular endothelial growth factor receptors (VGFR) such as ramucirumab has shown to significantly improve overall response rate (ORR), progression-free survival (PFS) and overall survival (OS) in patients with advanced gastric cancer (8). Furthermore, ramucirumab shows a better favorable risk profile compared to other anti-angiogenic agents and exhibits anti-angiogenic effects beyond progression (9).

Patients receiving first-line oxaliplatin regimens often develop neuropathy that may limit taxane tolerance or eligibility (10, 11). Because of concerns for taxane-related neuropathy as well as earlier exposure to taxane during the disease course in many patients, the identification of a taxane-free second-line therapy is of critical importance. A phase 3 clinical trial in colorectal cancer has shown safety and activity of RAM plus folinic acid (leucovorin), fluorouracil (5-FU), irinotecan and bevacizumab (FOLFIRI) as a second-line therapy after progression on folinic acid (leucovorin), 5-FU, and oxaliplatin with bevacizumab (12), thus providing a scientific basis for studying this combination in other cancers of the gastrointestinal tract. Despite the lack of large-scale randomized phase 3 trials, RAM plus FOLFIRI or irinotecan has emerged as a second-line option for patients with advanced GEA. The National Comprehensive Cancer Network (NCCN) guideline recommendations also support the use of RAM in combination with FOLFIRI or irinotecan as second-line therapy for patients with GEA.

In this literature review and meta-analysis, we aimed to identify publications, both clinical trials and retrospective studies, to review the data supporting the inclusion of RAM in combination with FOLFIRI or irinotecan as second-line therapy for patients with GEA and prior-taxane use as per the NCCN guideline recommendations; as well as reviewing safety data and performing an aggregated review to assess the efficacy of these combinations.

## 2 Methods

### 2.1 Literature searches

A systematic and comprehensive search of PubMed was performed to identify phase 2 clinical trials or retrospective studies using RAM plus FOLFIRI or irinotecan, including abstracts from major congresses, in addition to published manuscripts. The search was restricted to human studies, with no restrictions placed on language and all studies published before August 2022. The following search terms were combined: 1) gastric cancer OR gastric adenocarcinoma OR gastroesophageal junction cancer; 2) ramucirumab OR Cyramza; 3) FOLFIRI OR irinotecan, and 4) phase 2 clinical trial OR phase II clinical trial OR phase two clinical trial OR retrospective study. All results were reviewed and verified by the study team. All of the original publications were checked and reviewed; studies which were retrospective analyses or phase 2 clinical trials which examined the effectiveness and safety of RAM plus FOLFIRI or irinotecan were included for review. All manuscripts and publications that did not include the use of RAM plus FOLFIRI or irinotecan were excluded.

### 2.2 Statistical methodology

The ORR for each study was calculated with 95% confidence interval (CI) estimated from normal approximation. To generate the combined ORR with 95% CI, random-effects meta-analysis was conducted to synthesize response data from the available studies mentioned above. Logistic regression was used to model the binary response data with random effect accounting for across-study variability in the analysis. ORR in patients pretreated with taxane and patients who were taxane-naïve across studies were summarized with proportions and their 95% CI using an exact binomial approach.

In this review, only ORR data was pooled as the studies identified and reviewed had small sample sizes including both clinical trial studies and retrospective studies. Also, all studies reported ORR as the primary endpoint and not all studies reported PFS or OS data consistently which could provide clinically meaningful results.

## 3 Results

### 3.1 Literature searches

Six studies were identified, with nonoverlapping populations, 3 phase 2 clinical trials: Lorenzen et al. (13) (NCT03081143), Park et al. (14) (NCT03141034), and Kawamoto et al. (15) (UMIN000030372); and 3 retrospective studies: Klempner et al. (16), Vogl et al. (17), and Schlintl et al. (18). An overview of the studies is provided in **Table 1**. The baseline characteristics of patients in the studies identified are outlined in **Table 2**.

**Table 1:** Overview of studies included in review, including study type, treatment overview, overall response rate (ORR), median progression-free survival (PFS), and median overall survival (OS).

Study	Study Type	Clinical Trial Number	Treatment	Evaluable Patients	ORR	Median PFS (months)	Median OS (months)
Lorenzen et al. (13)	Phase 2 clinical trial (RAMIRIS)	NCT03081143	RAM plus FOLFIRI	72	22.2%	3.9	6.8
Park et al. (14)	Phase 2 clinical trial	NCT03141034	RAM plus Irinotecan	40*	29.0%	4.6	8.3
Kawamoto et al. (15)	Phase 2 clinical trial (HGCSG1603)	UMIN000030372	RAM plus Irinotecan	35**	25.9%	4.2	9.6
Klempner et al. (16)	Retrospective study		RAM plus FOLFIRI	26***	23.1%	6	13.4
Vogl et al. (17)	Retrospective study		RAM plus FOLFIRI	16^#^	23.1%	5.9	8.3
Schlintl et al. (18)	Retrospective study		RAM plus FOLFIRI or irinotecan	16	37.5%	5.4	7.6

*Park et al.: total patients enrolled N=40, for ORR, N=31 which included patients evaluable for radiographic response only. **Kawamoto et al.: Total patients enrolled N=35, for ORR N=27 patients included with at least one measurable lesion. ***Klempner et al.: Total 29 patients met the prespecified inclusion criteria, of which 26 were evaluable for PFS, OS and ORR analysis. ^#^Vogl et al. for ORR, N=13 response evaluable.

FOLFIRI, folinic acid (leucovorin), fluorouracil, and irinotecan; ORR, overall response rate; OS, overall survival; PFS, progression-free survival; RAM, ramucirumab.

**Table 2:** Baseline characteristics of patient cohorts receiving RAM plus FOLFIRI or RAM plus irinotecan.

Clinicopathologic feature	Lorenzen et al. (13) N=72		Park et al. (14) N=40		Kawamoto et al. (15) N=35		Klempner et al. (16)^#^ N=29		Vogl et al. (17) N=56*		Schlintl et al. (18) N=16	
Age, median (range), years	61		63 (27–81)		70 (47–80)		61.5 (36–80)		64 (38–82)		55 (46–71)	
Sex												
Male	47	65%	20	71%	25	71%	21	72%	36	64%	12	75%
Female	25	35%	8	29%	10	29%	8	28%	20	36%	4	25%
ECOG PS at second-line initiation												
0	30	42%	–	–	22	63%	12	45%	31	55%	2	13%
1	42	58%	–	–	13	37%	14	48%	–	–	10	63%
2	–	–	–	–	–	–	2	7%	25*	45%	4	25%
3	–	–	–	–	–	–	1	3%			–	–
Tumor location												
Esophagus	–	–	–	–	–	–	4	14%	–	–	–	–
GEJ	37	51%	18	64%	4	11%	8	28%	–	–	8	50%
Gastric	34	47%	10	36%	–	–	17	59%	26	46%	8	50%
AEG 1–3	–	–	–	–	–	–	–	–	30	54%	–	–
Stomach	–	–	–	–	31	89%	–	–	–	–	–	–
Lauren histology												
Diffuse	21	29%	–	–	13	37%	14	48%	39	70%	–	–
Intestinal	23	32%	–	–	17	49%	13	45%	6	11%	–	–
NOS	–	–	–	–	–	–	2	7%	–	–	–	–
Mixed	4	6%	–	–	5	14%	–	–	–	–	–	–
Metastatic disease sites												
Visceral	–	–	–	–	–	–	15	52%	–	–	–	–
Lymph node	35	49%	–	–	23	66%	20	70%	–	–	–	–
Radiographic peritoneal/peritoneum	25	35%	–	–	18	51%	13	45%	–	–	6	38%
Liver	29	40%	–	–	12	34%	–	–	–	–	–	–
Lung	10	14%	–	–	2	6%	–	–	–	–	–	–
Bone	14	19%	–	–	–	–	–	–	–	–	–	–
HER2 status at diagnosis												
IHC 0	–	–	–	–	–	–	17	55.0%	–	–	–	–
IHC 1+	–	–	–	–	–	–	4	13.0%	–	–	–	–
IHC 2+	–	–	–	–	–	–	4	13.0%	–	–	–	–
IHC 2+, FISH/NGS amp	–	–	–	–	–	–	1	–	–	–	–	–
IHC 2+, FISH/NGS non-amp	–	–	–	–	–	–	3	–	–	–	–	–
IHC 3+	–	–	–	–	–	–	1	3.0%	–	–	–	–
Negative	63	88.0%	23	82.0%	24	69.0%	–	–	–	–	–	–
Positive	7	10.0%	4	14.0%	9	26.0%	–	–	3.0	5.0%	–	–
Unknown/not tested	2	3.0%	1	4.0%	2	6.0%	2	7.0%	–	–	–	–
First-line therapy												
Docetaxel therapy	32	67.0%	–	–	–	–	–	–	–	–	–	–
S-1 + oxaliplatin	–	–	–	–	17	49.0%	–	–	–	–	–	–
Capecitabine + oxaliplatin	–	–	–	–	7	20.0%	–	–	–	–	–	–
FOLFOX	–	–	–	–	6	17.0%	–	–	7	12.5%	–	–
Nab-paclitaxel + S-1 + oxaliplatin	–	–	–	–	1	3.0%	–	–	–	–	–	–
Docetaxel + S-1 + CDDP	–	–	–	–	1	3.0%	–	–	–	–	–	–
S-1 + CDDP	–	–	–	–	1	3.0%	–	–	–	–	–	–
Capecitabine + CDDP	–	–	–	–	1	3.0%	–	–	–	–	–	–
S-1 + docetaxel	–	–	–	–	1	3.0%	–	–	–	–	–	–
FOLFOX/XELOX backbone	–	–	–	–	–	–	–	–	–	–	–	–
FOLFOX + trastuzumab	–	–	–	–	–	–	2	6.8%	–	–	–	–
FOLFOX + experimental agent (trial)	–	–	–	–	–	–	12	41.3%	–	–	–	–
Other 5-FU + platinum	–	–	–	–	–	–	6	20.7%	–	–	–	–
5-FU + platinum-containing triplets	–	–	–	–	–	–	6	20.7%	–	–	–	–
FLOT	–	–	–	–	–	–	3	10.3%	12	21.4%	–	–
DOF	–	–	–	–	–	–	1	3.4%	–	–	–	–
Modified DCF	–	–	–	–	–	–	1	3.4%	–	–	–	–
ECX + placebo/experimental agent (trial)	–	–	–	–	–	–	–	–	–	–	–	–
Cis- or carboplatin/5FU	–	–	–	–	–	–	–	–	–	–	–	–
DCF	–	–	–	–	–	–	–	–	–	–	–	–
CHT ± trastuzumab	–	–	–	–	–	–	–	–	–	–	–	–
Other	–	–	–	–	–	–	–	–	17	30.4%	–	–

*Vogl et al. baseline characteristics include all patients in study, not just patients treated with RAM plus FOLFIRI, for n=25 ECOG PS is denoted as >1 in the respective manuscript. ^#^Data from Klempner et al. for ethnicity: Hispanic/Latino (24%), White (55%), Black (10%), Asian (7%), and other (3%); stage at diagnosis: II (3%), III (21%), IV (76%); histological grade 1 (well differentiated) (3%), 2 (moderately differentiated) (21%), and 3 (poorly differentiated) (76%); Signet ring cell features: yes (48%), no (52%); Ascites yes (45%), no (55%). All data taken directly from papers, “–” indicates data not available for this clinicopathologic feature in this study.

5-FU, fluorouracil; AEG, esophagogastric junction; amp, amplification; CDDP, cisplatin; CHT, chemotherapy; DCF, Docetaxel, Cisplatin, and 5-fluorouracil; DOF, docetaxel, oxaliplatin, 5-fluorouracil; ECOG, Eastern Cooperative Oncology Group; ECX, epirubicin, cisplatin, and capecitabine; FISH, fluorescence in situ hybridization; FLOT, fluorouracil, leucovorin, oxaliplatin, and docetaxel; FOLFIRI, folinic acid (leucovorin), fluorouracil, and irinotecan; FOLFOX, folinic acid (leucovorin), fluorouracil, and oxaliplatin; GEA, gastroesophageal adenocarcinoma; GEJ, gastroesophageal junction; HER2, human epidermal growth factor receptor 2; IHC, immunohistochemistry; N, evaluable patients; NGS, next-generation sequencing; NOS, not otherwise specified; PS, performance status; RAM, ramucirumab; S-1, tegafur, oteracil, and gimeracil; XELOX, oxaliplatin and capecitabine.

### 3.2 Overview of outcomes for patients in clinical trials and retrospective studies

In the multicenter, randomized, phase 2 clinical trial by Lorenzen et al. (13) (RAMIRIS, NCT03081143) patients with GEA who progressed on 5-FU or platinum first-line treatment were randomized 2:1 to FOLFIRI plus RAM (Arm A, N=72) or RAM plus paclitaxel (Arm B, N=38). Patients treated with RAM plus FOLFIRI had a median OS of 6.8 months and a median PFS of 3.9 months. The 6-month OS rate in the FOLFIRI plus RAM arm was 54% (95% CI 44–67) and the study did not meet the primary endpoint for the comparison with historical control. There were 48 evaluable taxane-pretreated patients, with 12 responders (ORR, 25.0%) and 24 evaluable taxane-naïve patients, with 4 responders (ORR, 16.7%). Patients treated with RAM plus paclitaxel had a median OS of 7.6 months and a median PFS of 3.7 months.

In the single-arm, phase 2 study by Park et al. (14), (NCT03141034) 40 patients were enrolled. All patients received platinum-based chemotherapy prior to enrollment, 8 patients had human epidermal growth factor receptor 2-positive disease, and 6 patients had received an immune checkpoint inhibitor. Median PFS was 4.6 months (95% CI, 2.7–5.4). Of the 31 patients evaluable for response, 9 out of 30 patients evaluable for radiographic response only (29%) had objective responses (1 complete response, 8 partial responses) and 5 patients (16%) had stable disease greater than 6 months. There were 7 evaluable taxane-pretreated patients with 3 responders (ORR, 42.9%) and 26 evaluable taxane-naïve patients with 6 responders (ORR, 23.1%).

In the multi-institutional nonrandomized, single-arm, phase 2 clinical trial by Kawamoto et al. (15) (HGCSG1603; jRCTs011180029), 35 patients with advanced GEA who were refractory or intolerant to first-line chemotherapy were enrolled and treated with RAM plus irinotecan. Median PFS and OS were 4.2 months (95% CI, 2.5–5.4) and 9.6 months (95% CI, 6.4–16.6), respectively. Data from 27 patients with measurable disease (ORR, 25.9%) was used in the review and meta-analysis.

Klempner et al. (16) performed a retrospective study of 29 patients who had received second-line RAM plus FOLFIRI. In the 26 evaluable patients, median PFS was 6.0 months, with a range of 2 to 24 months, and median OS was 13.4 months.

Vogl et al. (17) performed a retrospective study of 56 patients treated with RAM plus paclitaxel (N=38, as second-line [75%] or beyond second-line [25%]) or RAM plus FOLFIRI (N=16). This study found a significant increase in the median PFS and OS of patients treated with RAM plus FOLFIRI compared with patients treated with RAM plus paclitaxel (P=0.05). The median PFS and OS for patients RAM plus paclitaxel was 2.9 months (95% CI, 2.3–3.6) and 4.4 months (95% CI, 4.1–4.7), respectively; for those treated with RAM plus FOLFIRI, the median PFS and OS was 5.9 months (95% CI, 0.4–11.4) and 8.3 months (95% CI, 6.6–10), respectively.

Schlintl et al. (18) performed a retrospective analysis of 16 patients with advanced or metastatic gastric cancer, who received treatment with RAM plus FOLFIRI or irinotecan. The median PFS and OS of all patients was 5.4 months (95% CI, 3.7–7.1) and 7.6 months (95% CI, 6.1–9.1), respectively. Patients receiving RAM plus FOLFIRI displayed a statistically significant longer OS compared with patients receiving RAM plus irinotecan, with a median of 15.2 months (95% CI, 4.7–25.7) versus 6.9 months (95% CI, 1.0–12.8; P=0.01), respectively. However, there was no statistically significant difference in the median PFS (5.4 versus 4.6 months, P=0.19).

**Figure 1:**
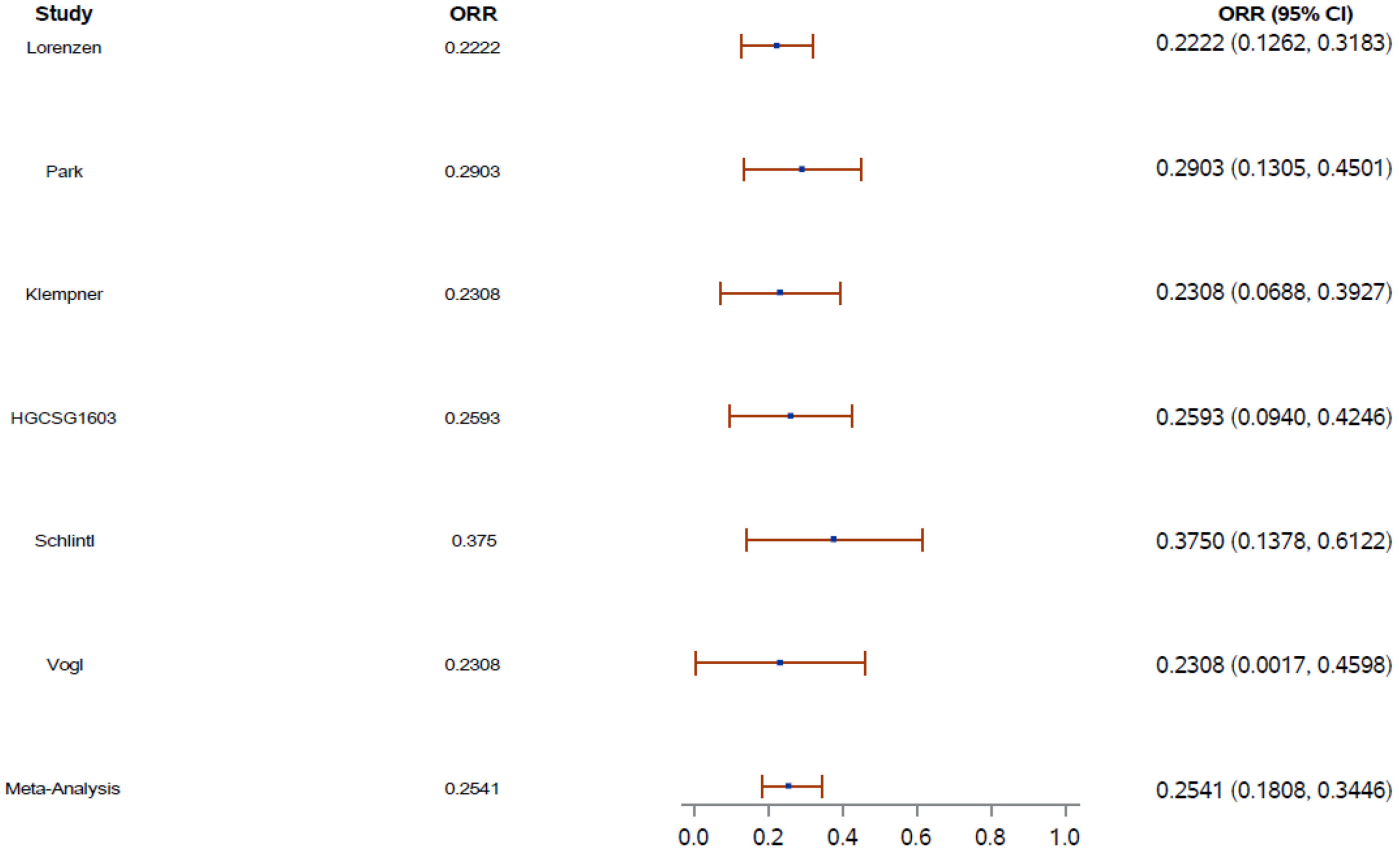
Overall response rate (ORR) aggregate weight (blue dot) on the basis of the number of patients in each study. Error shown as red bar. CI, confidence interval; ORR, overall response rate.

An aggregated review of ORR was performed using random-effects meta-analyses. The pooled ORR was 25.4% (95% CI, 18.0–34.5) (**Figure 1**).

### 3.3 Patients with prior-taxane use

Three studies were identified which included patients with prior-taxane use. These studies evaluated ORR and PFS in patients treated with RAM plus FOLFIRI.

In the studies by Lorenzen et al. (13), and Park et al. (14) there was a numerical increase in the ORR with RAM plus FOLFIRI in patients pretreated with taxane versus patients who were taxane-naïve (**Table 3**). The study by Klempner (16) observed an improved ORR in patients who were taxane-naïve versus patients who were pretreated with taxane.

**Table 3:** Table comparing the overall response rate (ORR) of patients pretreated with taxane versus patients who were taxane-naïve in the studies by Park et al., Lorenzen et al., and Klempner et al.

Study	Drug	Study Type	Taxane-pretreated			Taxane-naïve		
			Evaluable (n)	Responder (n)	ORR (95% CI)	Evaluable (n)	Responder (n)	ORR (95% CI)
Park et al. (14)	RAM plus Irinotecan	Phase 2	7	3	42.9% (6.2, 79.5)	26	6	23.1% (6.9, 39.3)
Lorenzen et al. (13)	RAM plus FOLFIRI	Phase 2	48	12	25.0% (12.8, 37.3)	24	4	16.7% (1.8, 31.6)
Klempner et al. (16)	RAM plus FOLFIRI	Retrospective	5	1	20.0% (0.0, 55.1)	23	6	26.1% (8.1, 44.0)

CI, confidence interval; FOLFIRI, folinic acid (leucovorin), fluorouracil, and irinotecan; n, patients in each category evaluable or responder; ORR, overall response rate RAM, ramucirumab.

In the study by Lorenzen et al. (13), for patients with prior docetaxel treatment (72/110), the median PFS was 4.6 months for patients treated with RAM plus FOLFIRI versus 2.1 months for patients treated with RAM plus paclitaxel, and the median OS was 7.5 months versus 6.6 months, respectively. Sixty-seven patients were evaluable for response and were pretreated with docetaxel. ORR was 25% in patients treated with RAM plus FOLFIRI and 8% in patients treated with RAM plus paclitaxel. Disease control rate was 65% and 38% for RAM plus FOLFIRI and RAM plus paclitaxel, respectively.

Vogl et al. (17) observed a trend towards prolonged PFS after perioperative taxane-based 5-FU, leucovorin, oxaliplatin, and docetaxel chemotherapy (N=12) with RAM plus FOLFIRI compared with RAM plus paclitaxel, with a median PFS of 5.6 months (95% CI, 4–7.8) and 2.9 months (95% CI, 1.6–4.3), respectively. In data from the study by Klempner et al. (16), there was an improved ORR (maximum partial responses) for patients who were taxane-naïve (partial response, 44.8%) versus patients who were pretreated with taxane (partial response, 20.7%). This may be because of the low number of patients pretreated with taxane included in the retrospective study.

### 3.4 Safety

The safety profile reported across all reviewed studies showed that with RAM plus FOLFIRI or irinotecan, the most common adverse event (AE) at any grade was neutropenia. In the clinical trial by Lorenzen et al. (13), the most common grade ≥3 AEs in patients treated with RAM plus FOLFIRI were neutropenia (N=12, 17%), leukopenia (N=3, 4%), diarrhea (N=7, 10%), and stomatitis (N=7, 10%). Of the patients treated with RAM plus FOLFIRI, 56% had at least 1 serious AE. In the study by Kawamoto et al. (15), the most common grade ≥3 AEs were neutropenia (N=18, 51%), leukopenia (N=15, 43%), anemia (N=7, 20%), anorexia (N=5, 14%), and febrile neutropenia (N=4, 11%). No deaths or new safety signals with a causal relation to the study treatment were observed. In the study by Park et al. (14), diarrhea (N=27, 68%), nausea (N=24, 60%), vomiting (N=18, 45%), and neutropenia (N=15, 38%) were common AEs; no grade 3 or 4 neuropathy was reported.

In the retrospective studies, Klempner et al. (16) found toxicities were largely grade 1 or 2, with only 6.9% developing grade 3 or 4 AEs (all fatigue, grade 3). Fatigue (76%), diarrhea (31%), anemia (24%), and neutropenia (14%) were the most common AEs, and there were no toxic deaths. Vogl et al. (17) found the most common grade 3 toxicity for patients treated with RAM plus FOLFIRI was neutropenia (44%), followed by diarrhea, fatigue, and polyneuropathy. Safety data were not available for the study by Schlintl et al. (18), however, only 1 patient discontinued RAM-based therapy because of toxicity.

## 4 Discussion

As per NCCN guidelines, oxaliplatin-based regimens are generally preferred over cisplatin-based regimens as first-line therapy for locally advanced, recurrent, or metastatic gastric cancer. The preferred second-line therapy regimens include ramucirumab and paclitaxel, fam-trastuzumab deruxtecan-nxki for HER2 over expressive positive adenocarcinoma, docetaxel, paclitaxel, irinotecan, fluorouracil and irinotecan, and trifluridine and tipiracil for third-line or subsequent therapy. Careful consideration must be given when selecting a second-line therapy, particularly for safety, efficacy, and treatment compliance.

Ramucirumab plus FOLFIRI or irinotecan is a non-neurotoxic regimen comparing favorably with the combination of RAM plus paclitaxel used in the seminal RAINBOW trial (6). In this review, we examined multiple prospective phase 2 clinical trials and retrospective studies to analyze the data supporting RAM plus FOLFIRI or irinotecan as second-line therapy for patients with GEA. While the number of evaluable patients varied across these studies, ORR ranged from 22% to 38%, median PFS ranged from 3.9 to 6.0 months, and median OS ranged from 6.8 to 13.4 months.

The initial results from the phase 2 clinical trial by Lorenzen et al. provided a rationale for continuation of the trial as phase 3, which enrolls patients who were pretreated with taxane only and is currently recruiting (13, 19). Data reported by Park et al. and Kawamoto et al. demonstrated comparable efficacy outcomes as observed by Lorenzen et al. (13–15).

Vogl et al. (17) found that RAM plus FOLFIRI-treated patients showed favorable results with a better median PFS than RAM plus paclitaxel-treated patients (P=0.05). This highlights the potential for RAM plus FOLFIRI or irinotecan combinations as an alternative to treatment with taxanes, fulfilling a huge unmet clinical need for GEA patients. However, studies by both Lorenzen et al. and Vogl et al. showed that patients pretreated with taxanes had better outcomes when treated with RAM plus FOLFIRI combination than when treated with paclitaxel (13, 17). Overall, 2 of the 3 studies reported better ORR in patients pretreated with taxanes followed by RAM plus FOLFIRI. However, given the small sample sizes, the retrospective design and overlapping confidence intervals, no conclusions can be drawn from these results.

Further support for safety of RAM plus irinotecan as second-line therapy was also shown in a small phase 1b (N=6) Japanese trial (20). The authors found this regimen was well tolerated by patients with advanced gastric cancer. In addition, the RAISE trial with a large sample size of over 500 patients with metastatic colorectal cancer (progressed on or after first-line oxaliplatin-based therapy) treated with second-line RAM plus FOLFIRI showed that RAM plus FOLFIRI resulted in improved OS and was well tolerated with no new safety findings (12). Overall, treatment with RAM plus FOLFIRI or irinotecan was well tolerated by patients. The most common AE of any grade observed was netropenia, which is in line with RAM toxicity profiles known from FOLFIRI or irinotecan regimens (**Table 4**).

**Table 4:** Available toxicity profiles of cohorts of patients with advanced GEA receiving second-line RAM plus FOLFIRI or irinotecan, N (%).

Adverse Events of Interest	Lorenzen et al. (13) (N=72)	Park et al. (14) (N=40)	Kawamoto et al. (15) (N=35)	Klempner et al. (16) (N=29)	Vogl et al. (17) (N=16)
Diarrhea grade 1/2	22 (31.0%)	24 (60.0%)	14 (40.0%)	9 (31.0%)	<44.0%
Diarrhea grade 3/4	7 (10.0%)	3 (8.0%)	3 (9.0%)		
Anemia grade 1/2	15 (21.0%)	29 (73.0%)	22 (63.0%)	7 (24.1%)	
Anemia grade 3/4	5 (7.0%)	1 (3.0%)	7 (20.0%)		
Neutropenia, any grade	21 (29.0%)	15 (38.0%)	29 (83.0%)	4 (13.8%)	44.0%
Fatigue grade 1/2	29 (40.0%)	24 (60.0%)	26 (66.0%)	20 (69.0%)	Yes
Fatigue grade >2	3 (4.0%)	4 (10.0%)	1 (3.0%)	2 (6.9%)	Yes
Hypertension grade 1/2	4 (6.0%)	7 (18.0%)	29 (83.0%)	2 (6.9%)	7.0%
Hypertension grade 3/4	1 (1.0%)	7 (18.0%)	3 (9.0%)		
Anorexia grade 1/2		16 (40.0%)	17 (49.0%)		
Anorexia grade 3/4		1 (3.0%)	5 (14.0%)		
Leukopenia 1/2	10 (14.0%)	19 (48.0%)	12 (34.0%)		
Leukopenia 3/4	3 (4.0%)	4 (10.0%)	15 (43.0%)		
Stomatitis all grades	16 (22.0%)				

Among the 6 studies reviewed, 5 reported safety results and Schlintl et al. did not report safety.

FOLFIRI, folinic acid (leucovorin), fluorouracil, and irinotecan; GEA, gastroesophageal adenocarcinoma; N, total number of patients in each study; RAM, ramucirumab.

The culmination of the available data to date, including work published by Klempner et al. (16), has resulted in the inclusion of RAM plus FOLFIRI or irinotecan in the NCCN Clinical Practice Guidelines for second-line treatment of GEA (3).

When analyzing these data, a number of additional factors should be considered, including duration of neuropathy, grade, resolution, and other comorbidities that can affect second-line efficacy outcomes. Additionally, the time between prior treatment (both taxane-pretreated and taxane-naïve) and FOLFIRI or irinotecan with RAM should be considered when determining the differences in effectiveness (ORR).

Given this, it is not possible to establish why differences in ORR are observed without speculation. In the study by Lorenzen et al. (13), a numerical increase in ORR was observed for patients who were pretreated with a taxane, however, these results are inconclusive given the small sample size.

Limitations of this study include the small sample sizes in the studies reviewed, and limited availability of data presented at congresses for some of the reports. The reviewed studies differed with respect to study design, eligibility, and response criteria. In addition, the studies were not designed to determine statistical differences in efficacy endpoints on the basis of prior-taxane versus naïve-taxane patient groups. Also, the studies reviewed did not have consistent RAM plus FOLFIRI or RAM plus irinotecan as comparator arms. A few studies had paclitaxel plus RAM as the comparator. To determine the benefit of the alternative strategies and make a definitive conclusion on RAM-based treatment regimens, the ideal comparator arm would be RAM plus FOLFIRI or RAM plus irinotecan. Despite the limitations, there are noteworthy strengths of this review such as the patients across the reviewed studies include a more representative patient sample, the baseline characteristics were generally consistent across all studies, and the patients across the reviewed studies were inclusive of multiple geographies. The first-line treatment landscape has evolved with recent approvals of CheckMate-649, KEYNOTE-590, and KEYNOTE-811 involving PD-1 inhibitor therapeutic options. With the utilization of frontline immune checkpoint inhibition regimens, the efficacy of subsequent RAM combinations remains an important consideration in treatment sequencing strategies. In a retrospective analysis, Sasaki et al. reported better efficacy in patients receiving RAM plus taxanes when exposed to prior anti-PD-1 treatments as compared with the reversed sequence (21). Similar data were presented by Kankeu Fonkoua et al. (22, 23) demonstrating predefined serial immunotherapy combinations followed by RAM plus taxanes provides efficacy benefits and may overcome resistance to PD-1 inhibitor therapy. An ongoing study is expected to further analyze these findings in a prospective setting (SEQUEL [NCT04069273]). Also, as noted earlier, there is an ongoing phase 2 RAMIRIS clinical trial, assessing the efficacy and safety of RAM plus FOLFIRI versus RAM plus paclitaxel in patients with previous taxane therapy (NCT03081143) which will provide additional data and further evidence.

The studies identified in this review suggest that patients previously treated with systemic therapy maintains benefits with RAM-based treatment regimens irrespective of prior-taxane use. This treatment strategy will especially benefit patients who become ineligible to receive RAM plus paclitaxel. Also, RAM-based treatment regimens are included in NCCN category 2A (lower levels of evidence, uniform expert opinion) recommendations.

While this review supports the safety and clinical benefit of RAM plus FOLFIRI or irinotecan combination on the basis of small clinical trials and retrospective analyses, a randomized phase 3 study would provide stronger evidence. Results from phase 3 trials and additional data are needed to provide additional evidence.

## Data Availability

The original contributions presented in the study are included in the article/supplementary material. Further inquiries can be directed to the corresponding author.
